# Supplementary material for: Structural clustering across the tree of life reveals candidate Asgard–eukaryote homologs and a shared expansion of solenoids
Source: bioRxiv. 2026 Oct 29:2026.10.28.762692. Preprint. [Version 1] doi: 10.64898/2026.10.28.762692 (PMC13734099; doi:10.64898/2026.10.28.762692)
Supplement: Supplementary file 1 [file NIHPP2026.10.28.762692v1-supplement-3.pdf]

bioRxiv preprint doi: https://doi.org/10.64898/2026.10.28.762692; this version posted October 29, 2026. The copyright holder for this preprint (which was not certified by peer review) is the author/funder, who has granted bioRxiv a license to display the preprint in perpetuity. It is made available under a CC-BY 4.0 International license.

Supplementary Figures & Tables

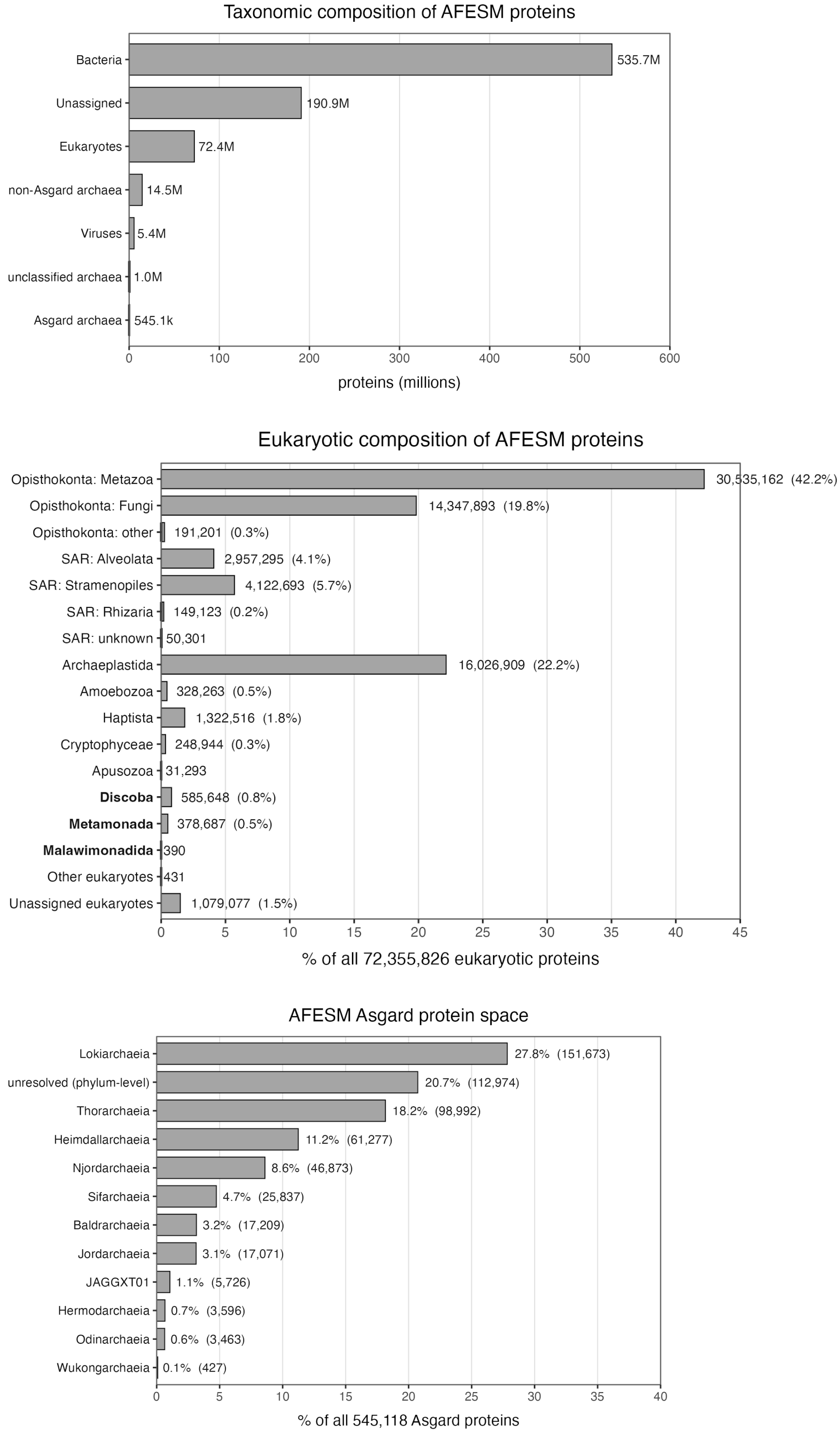

Supplementary Figure 1. Taxonomic distribution of protein entries in the AFESM database. Distribution across major taxonomic groups (A), eukaryotic supergroups, and Asgard archaeal classes (C).

bioRxiv preprint doi: https://doi.org/10.64898/2026.10.28.762692; this version posted October 29, 2026. The copyright holder for this preprint (which was not certified by peer review) is the author/funder, who has granted bioRxiv a license to display the preprint in perpetuity. It is made available under a CC-BY 4.0 International license.

Table S1: Robustness of the taxonomic composition of AFESM structural clusters to alternative taxonomic-presence criteria.

Presence criterion	Bacteria-only	Multi-domain	Eukaryote-only	Archaea-only	Unassigned-and/or viral-only	**Asgard+euk (ESP candidates)**
≥1 protein	1,098,343 (58.0%)	620,233 (32.7%)	138,506 (7.3%)	19,348 (1.0%)	18,284 (1.0%)	299 (0.016%)
≥2 proteins	1,242,525 (65.6%)	437,269 (23.1%)	161,216 (8.5%)	24,745 (1.3%)	28,959 (1.5%)	232 (0.012%)
≥1% of proteins	1,198,343 (63.2%)	507,542 (26.8%)	149,238 (7.9%)	21,013 (1.1%)	18,578 (1.0%)	289 (0.015%)
≥2 proteins & ≥1%	1,287,891 (68.0%)	387,083 (20.4%)	165,357 (8.7%)	25,377 (1.3%)	29,006 (1.5%)	224 (0.012%)

Values are the number of clusters, with percentages calculated relative to all 1,894,714 clusters. Taxonomic presence was evaluated using four criteria: at least one sequence, at least two sequences, at least 1% of all cluster members, or both at least two sequences and at least 1% of all cluster members. "Asgard + eukaryote" clusters are a subset of the multi-domain category and are reported separately because they represent candidate ESP families. The unassigned/viral-only category comprises clusters containing exclusively metagenomic unassigned proteins, viral proteins, or both. The overall taxonomic composition remained similar across all four criteria.

bioRxiv preprint doi: https://doi.org/10.64898/2026.10.28.762692; this version posted October 29, 2026. The copyright holder for this preprint (which was not certified by peer review) is the author/funder, who has granted bioRxiv a license to display the preprint in perpetuity. It is made available under a CC-BY 4.0 International license.

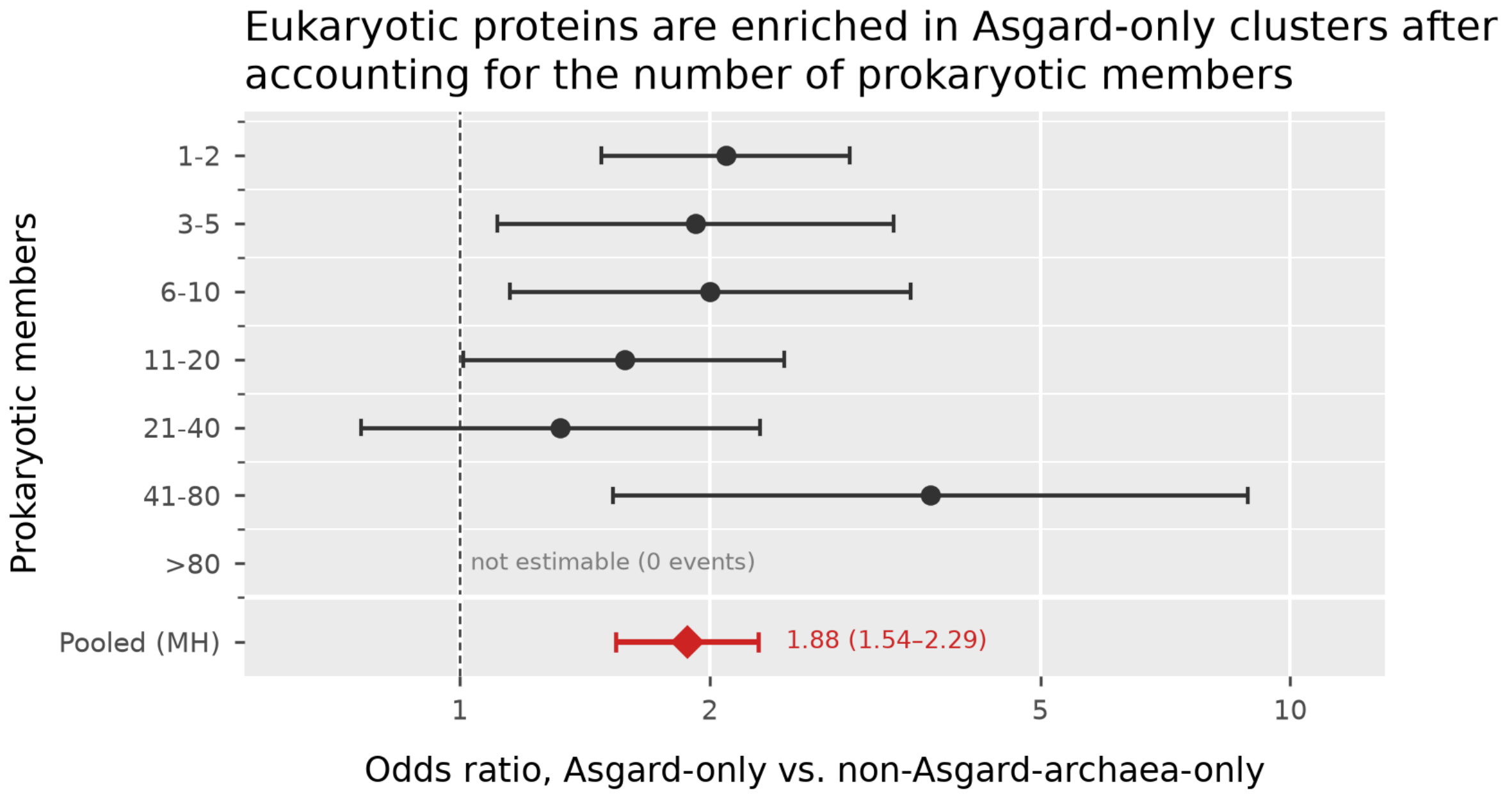

Supplementary Figure 2. Eukaryotic proteins remain enriched in clusters with exclusively Asgard rather than non-Asgard prokaryotic members, even after accounting for the number of prokaryotic members. Clusters were classified as Asgard-only or non-Asgard-only according to whether their prokaryotic members originated exclusively from Asgard or non-Asgard archaea, respectively. They were then grouped according to their number of prokaryotic members. Black circles and horizontal bars show the odds ratios and 95% confidence intervals for each group; the red diamond shows the pooled Mantel–Haenszel estimate. The dashed vertical line at an odds ratio of 1 marks no difference between Asgard-only and non-Asgard-only clusters. After adjustment for the number of prokaryotic members, clusters containing exclusively Asgard proteins as prokaryotic members remained more likely to contain eukaryotic proteins than those containing exclusively non-Asgard archaeal proteins as prokaryotic members (pooled odds ratio = 1.88, 95% CI 1.54–2.29). The corresponding unadjusted odds ratio was 2.69 (95% CI 2.33–3.10).

bioRxiv preprint doi: https://doi.org/10.64898/2026.10.28.762692; this version posted October 29, 2026. The copyright holder for this preprint (which was not certified by peer review) is the author/funder, who has granted bioRxiv a license to display the preprint in perpetuity. It is made available under a CC-BY 4.0 International license.

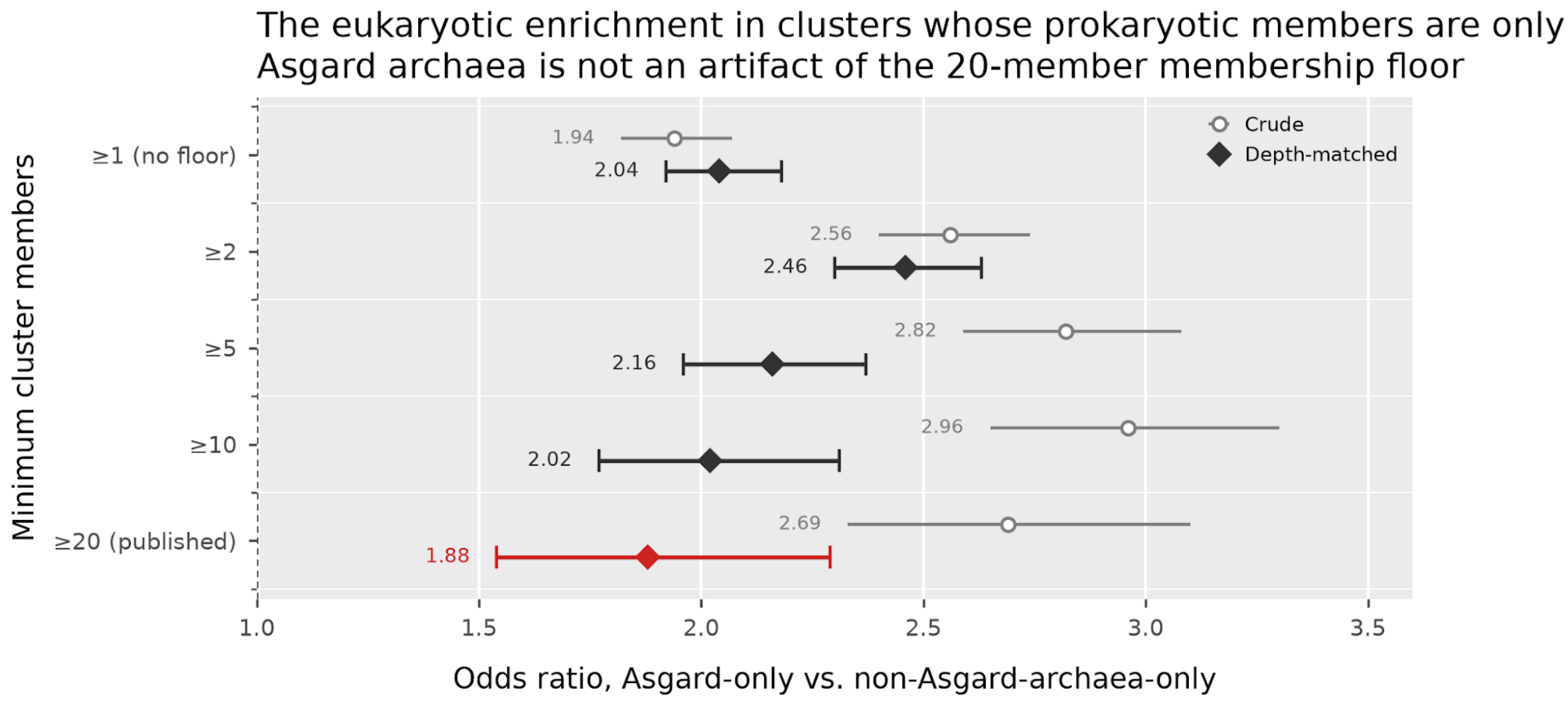

Supplementary Figure 3. Eukaryotic proteins remain enriched in clusters whose only prokaryotic members are Asgard archaea independently of the 20-member membership floor. Structural clusters whose prokaryotic members derive exclusively from Asgard archaea were compared with those deriving exclusively from non-Asgard archaea, at five minimum cluster sizes. Open grey circles show crude odds ratios for the presence of eukaryotic members; filled diamonds show Mantel-Haenszel odds ratios pooled over seven strata of prokaryotic membership (1-2, 3-5, 6-10, 11-20, 21-40, 41-80 and >80 members); horizontal bars are 95% confidence intervals and the dashed line marks an odds ratio of 1. The red diamond is the main analysis 20-member floor.

bioRxiv preprint doi: https://doi.org/10.64898/2026.10.28.762692; this version posted October 29, 2026. The copyright holder for this preprint (which was not certified by peer review) is the author/funder, who has granted bioRxiv a license to display the preprint in perpetuity. It is made available under a CC-BY 4.0 International license.

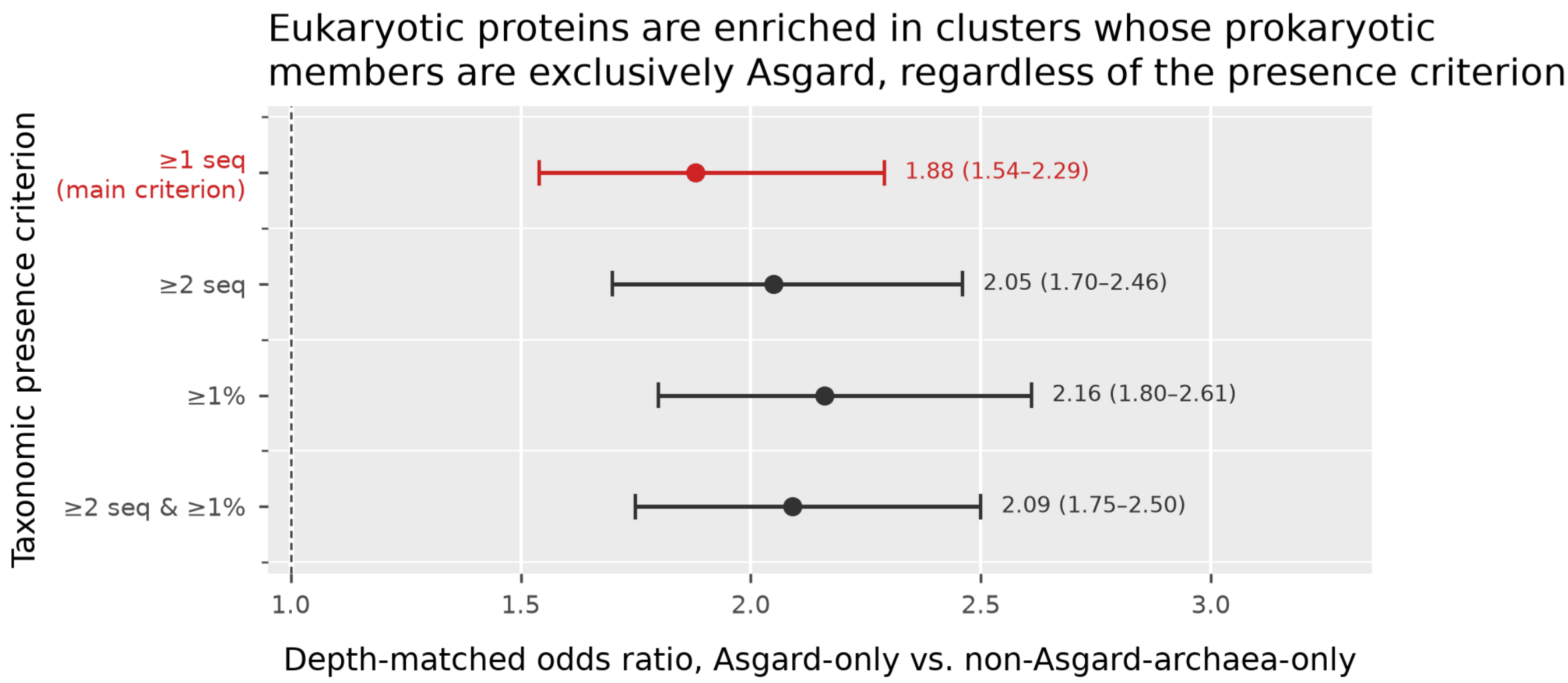

Supplementary Figure 4: Clusters whose prokaryotic members came exclusively from Asgard archaea were more likely to contain eukaryotic proteins than those whose prokaryotic members came exclusively from non-Asgard archaea, under all definitions of taxonomic presence. The analysis, restricted to clusters containing at least 20 proteins, was repeated using four definitions of taxonomic presence: ≥1 sequence (main criteria), ≥2 sequences, ≥1% of all sequences in the cluster, or both ≥2 sequences and ≥1%. Under each criterion, the classification of clusters as Asgard-only or non-Asgard-only and the presence of eukaryotic proteins were reassessed. To prevent cluster size from influencing the results, clusters with similar numbers of prokaryotic proteins were compared (an adjustment referred to as depth correction). Diamonds show the estimated odds ratios, and horizontal bars show the corresponding confidence intervals. Values greater than 1 indicate that eukaryotic proteins were more likely to occur in Asgard-only clusters.

bioRxiv preprint doi: https://doi.org/10.64898/2026.10.28.762692; this version posted October 29, 2026. The copyright holder for this preprint (which was not certified by peer review) is the author/funder, who has granted bioRxiv a license to display the preprint in perpetuity. It is made available under a CC-BY 4.0 International license.

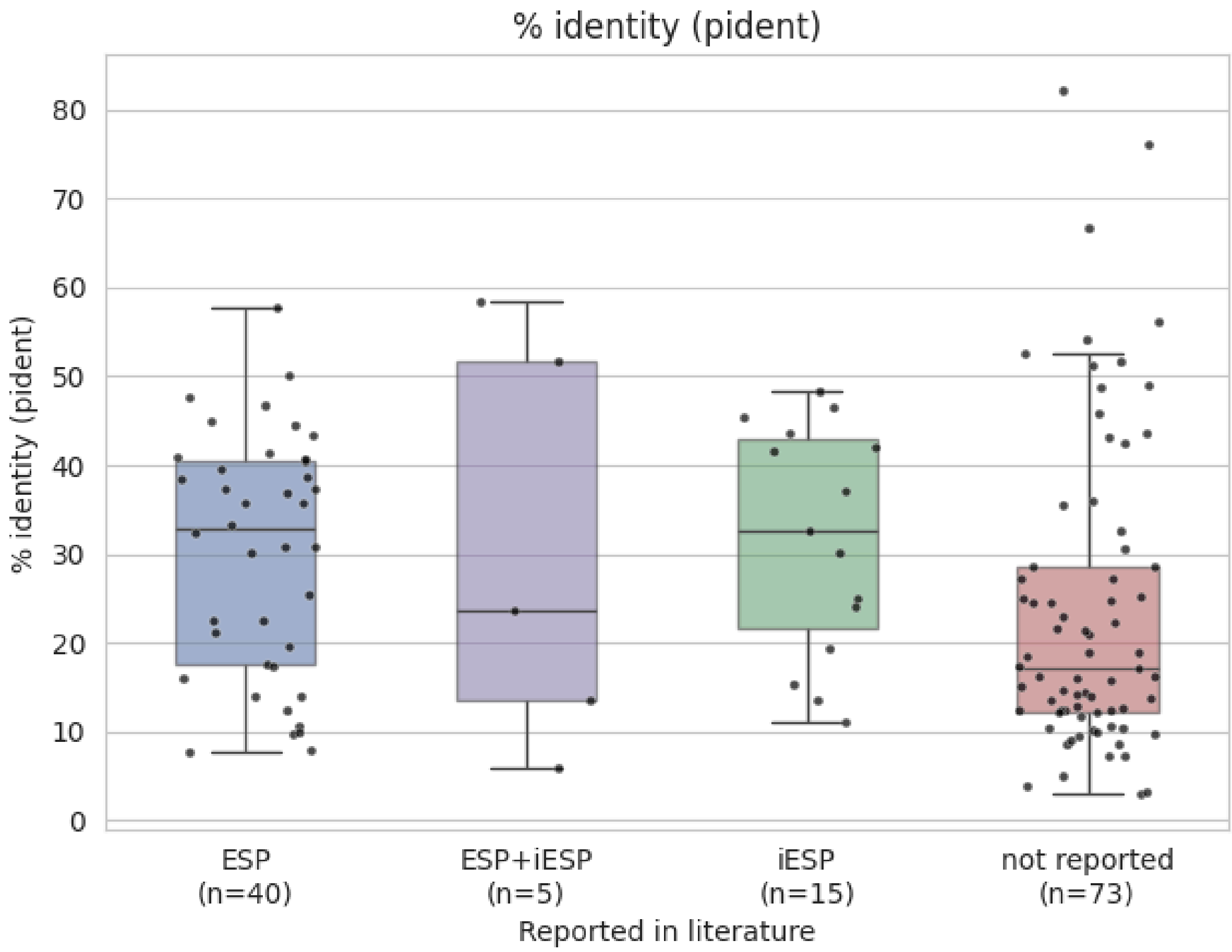

Supplementary Figure 5. Sequence identity between Asgard and eukaryotic members of structural clusters, by match to the previously reported eukaryotic signature protein (ESP) catalog. Maximum pairwise amino acid identity between Asgard and eukaryotic members of each of the 133 Asgard–eukaryote structural clusters. Clusters are grouped by match to the ESP catalog of Köstlbacher et al. (2026). The catalog combines ESPs identified with sequence-domain profiles (Eme et al. 2023; Liu et al. 2021) and ESPs identified by structural similarity, which the authors termed isomorphic ESPs (iESPs). Groups are: no catalog match (n = 73), sequence-defined ESPs only (n = 40), iESPs only (n = 15), and both ESP/iESP (n = 5). Identities are the highest values observed in Foldseek alignments between Asgard and eukaryotic members of each cluster. Boxes show the interquartile range, centre lines the median, and whiskers 1.5× the interquartile range; points are individual clusters.

bioRxiv preprint doi: https://doi.org/10.64898/2026.10.28.762692; this version posted October 29, 2026. The copyright holder for this preprint (which was not certified by peer review) is the author/funder, who has granted bioRxiv a license to display the preprint in perpetuity. It is made available under a CC-BY 4.0 International license.

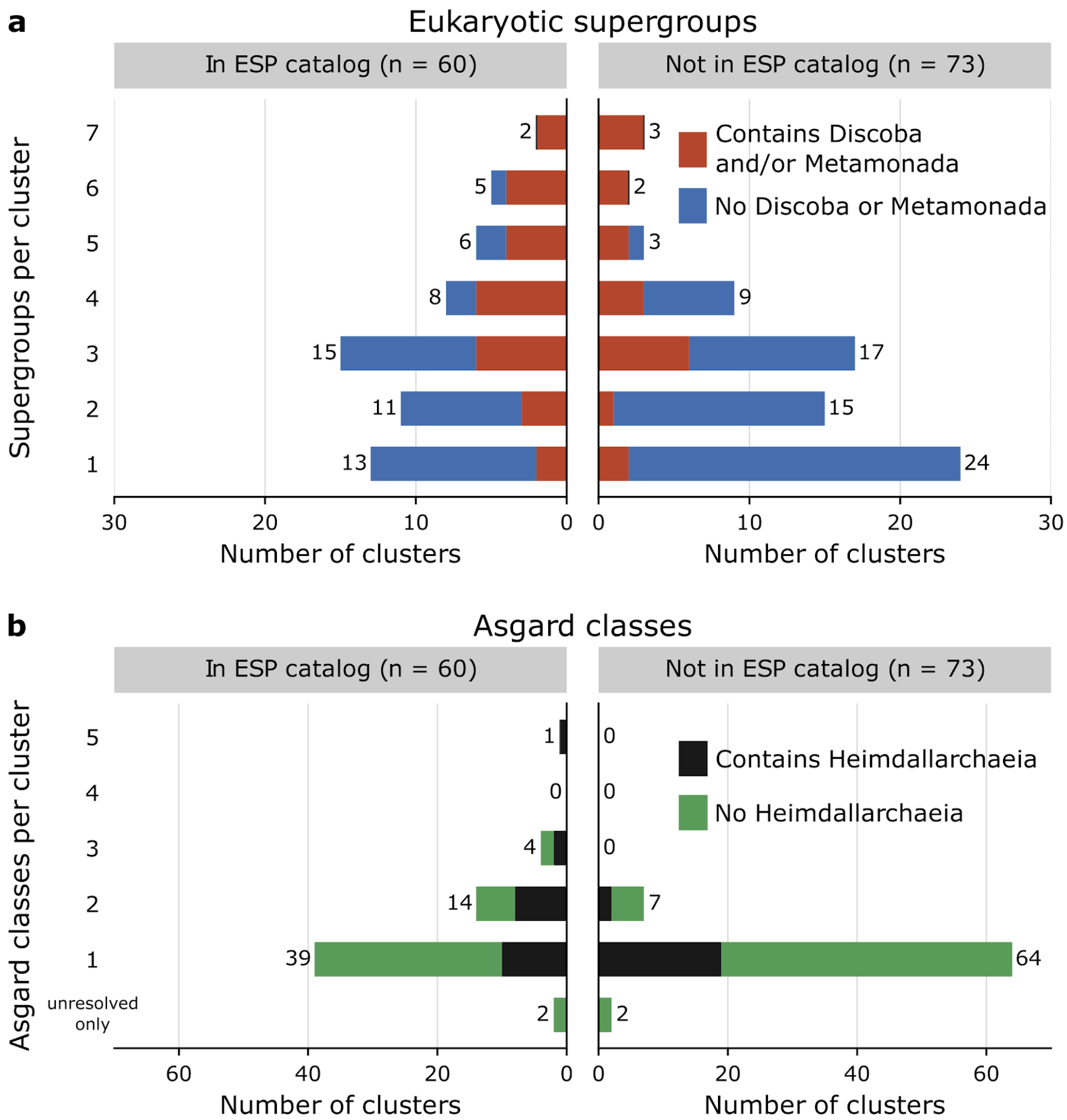

Supplementary Figure 6. Distribution of eukaryotic supergroups (a) and Asgard classes (b) across Asgard–eukaryote structural clusters, split by whether the cluster contains a previously reported ESP. The 133 clusters containing ≥2 Asgard and ≥2 eukaryotic proteins were classified as 'In ESP catalog' (n = 60) when at least one Asgard member matched an entry of the ESP reference catalog (Köstlbacher et al. 2026), and as 'Not in ESP catalog' (n = 73) otherwise. a) Number of eukaryotic supergroups represented per cluster. Bars are coloured by whether the cluster includes Discoba and/or Metamonada, lineages which are placed within the two distinct multi-group assemblages on either side of the eukaryotic root (Williamson et al. 2025) (red), or neither (blue). b) Number of Asgard classes represented per cluster. Bars are coloured by whether the cluster includes Heimdallarchaeia, the Asgard class containing the closest known relatives of eukaryotes (Eme et al. 2023; Zhang et al. 2025) (black), or not (green). 'Unresolved only' denotes clusters whose Asgard members could not be assigned to a class. Numbers next to the bars give the total number of clusters in each category. In each panel, the bars of each half sum to that half's n.

bioRxiv preprint doi: https://doi.org/10.64898/2026.10.28.762692; this version posted October 29, 2026. The copyright holder for this preprint (which was not certified by peer review) is the author/funder, who has granted bioRxiv a license to display the preprint in perpetuity. It is made available under a CC-BY 4.0 International license.

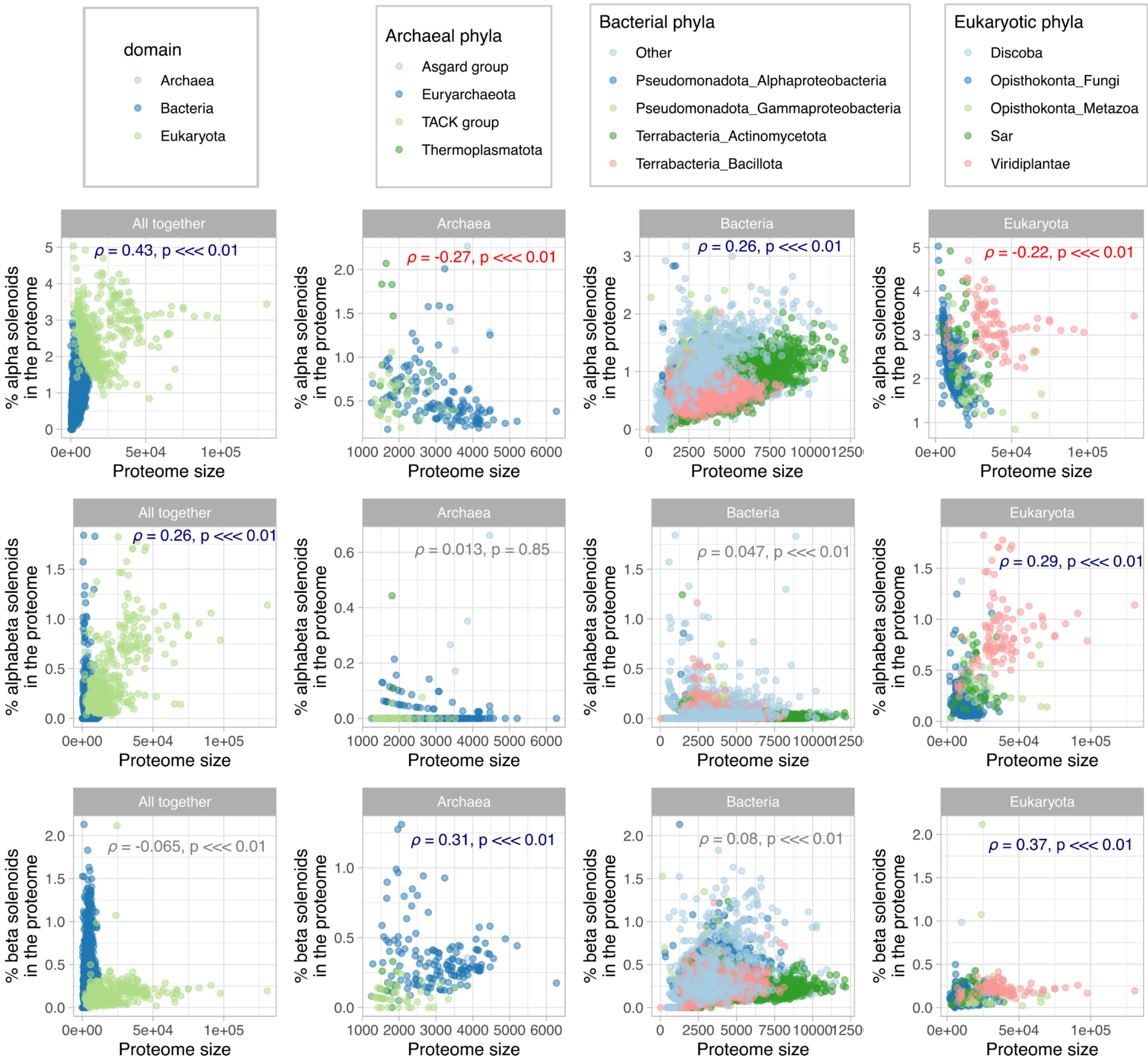

Supplementary Figure 7: Correlation analyses between solenoid content and proteome size across the tree of life. Scatter plots show the proportion of α-, αβ-, and β-solenoids (rows) in complete proteomes from AFDB, plotted against proteome size, separated by domain of life (columns) and colored by major lineages. Spearman's ρ and p-values are indicated; due to large sample sizes, some correlations are statistically significant but effect sizes are weak (ρ < 0.1).

bioRxiv preprint doi: https://doi.org/10.64898/2026.10.28.762692; this version posted October 29, 2026. The copyright holder for this preprint (which was not certified by peer review) is the author/funder, who has granted bioRxiv a license to display the preprint in perpetuity. It is made available under a CC-BY 4.0 International license.

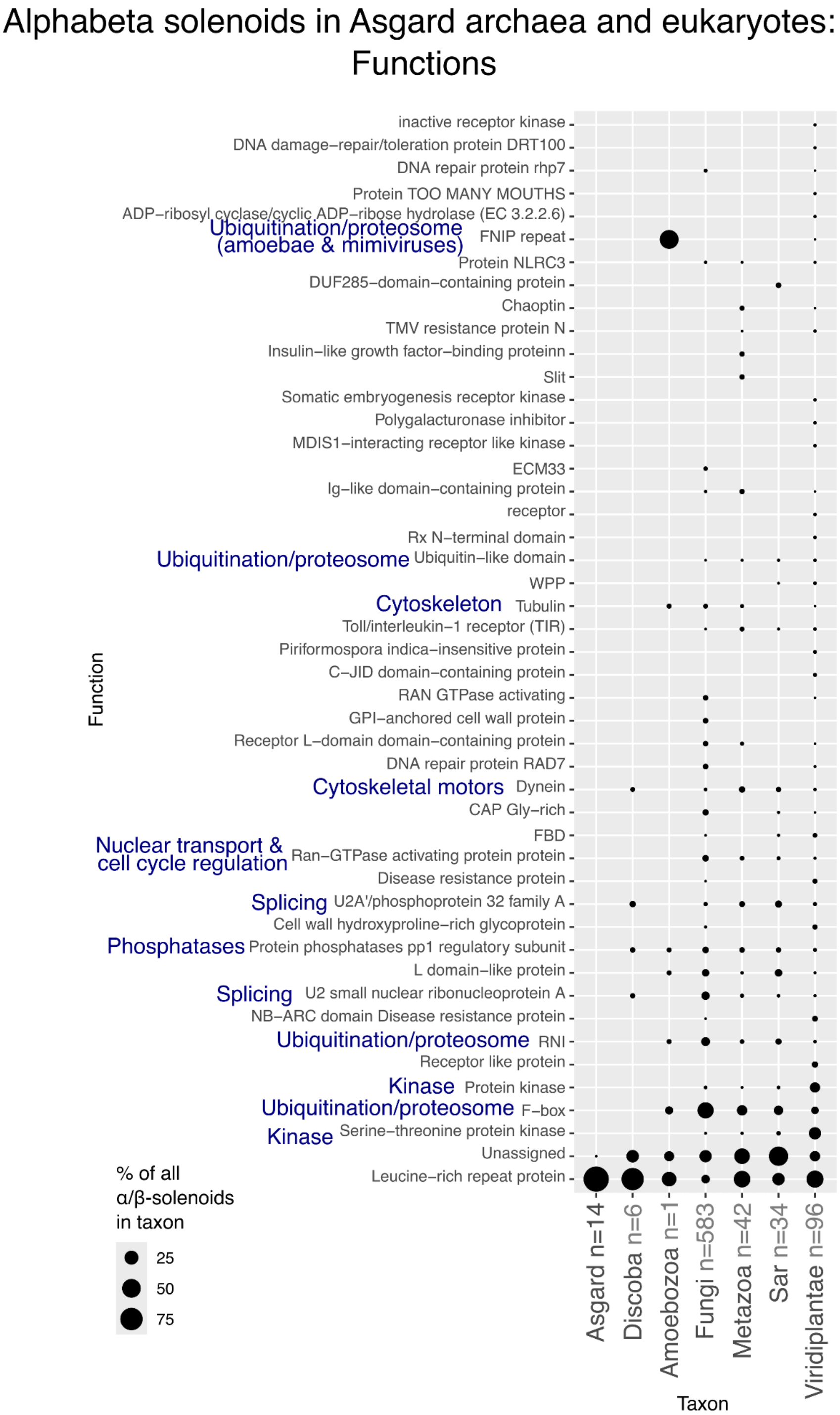

Supplementary Figure 8: Functional annotation of αβ solenoids in Asgard archaeal and eukaryotic proteomes. Functional categories (y-axis) were assigned from UniProt annotations. Circle size is proportional to the percentage of αβ solenoids in each taxonomic group (x-axis) associated with a given functional category (y-axis). The number of proteomes analyzed per taxon is indicated in the x-axis labels. Asgard αβ solenoids are frequently unannotated or broadly classified (e.g., “leucine-rich repeats”), reflecting the difficulty of inferring precise functions for this versatile structural fold. In contrast, many eukaryotic αβ solenoids are associated with hallmark eukaryotic pathways (highlighted in blue).

bioRxiv preprint doi: https://doi.org/10.64898/2026.10.28.762692; this version posted October 29, 2026. The copyright holder for this preprint (which was not certified by peer review) is the author/funder, who has granted bioRxiv a license to display the preprint in perpetuity. It is made available under a CC-BY 4.0 International license.

References

Akıl, Caner, Samson Ali, Linh T. Tran, et al. 2022. "Structure and Dynamics of Odinarchaeota Tubulin and the Implications for Eukaryotic Microtubule Evolution." *Science Advances* 8 (12): eabm2225.

Akıl, Caner, and Robert C. Robinson. 2018. "Genomes of Asgard Archaea Encode Profilins That Regulate Actin." *Nature* 562 (7727): 439–443.

Akıl, Caner, Linh T. Tran, Magali Orhant-Prioux, et al. 2020. "Insights into the Evolution of Regulated Actin Dynamics via Characterization of Primitive Gelsolin/cofilin Proteins from Asgard Archaea." *Proceedings of the National Academy of Sciences of the United States of America* 117 (33): 19904–19913.

Baum, David A., and Buzz Baum. 2014. "An inside-out Origin for the Eukaryotic Cell." *BMC Biology* 12 (October): 76.

Benjamini, Yoav, and Yosef Hochberg. 1995. "Controlling the False Discovery Rate: A Practical and Powerful Approach to Multiple Testing." *Journal of the Royal Statistical Society. Series B, Statistical Methodology* 57 (1): 289–300.

Breslow, N. E., and N. E. Day. 1980. "Statistical Methods in Cancer Research. Volume I - The Analysis of Case-Control Studies." *IARC Scientific Publications*, no. 32: 5–338.

Devos, Damien, Svetlana Dokudovskaya, Frank Alber, et al. 2004. "Components of Coated Vesicles and Nuclear Pore Complexes Share a Common Molecular Architecture." *PLoS Biology* 2 (12): e380.

Dohmen, Elias, Steffen Klasberg, Erich Bornberg-Bauer, Sören Perrey, and Carsten Kemena. 2020. "The Modular Nature of Protein Evolution: Domain Rearrangement Rates across Eukaryotic Life." *BMC Evolutionary Biology* 20 (1): 30.

Donoghue, Philip C. J., Chris Kay, Anja Spang, et al. 2023. "Defining Eukaryotes to Dissect Eukaryogenesis." *Current Biology : CB* 33 (17): R919–R929.

Edgar, Robert C., and Harutyun Sahakyan. 2025. "Protein Structure Alignment Significance Is Often Exaggerated." In *bioRxiv*. August 17. https://doi.org/10.1101/2025.07.17.665375.

Eme, Laura, Susan C. Sharpe, Matthew W. Brown, and Andrew J. Roger. 2014. "On the Age of Eukaryotes: Evaluating Evidence from Fossils and Molecular Clocks." *Cold Spring Harbor Perspectives in Biology* 6 (8). https://doi.org/10.1101/cshperspect.a016139.

Eme, Laura, Daniel Tamarit, Eva F. Caceres, et al. 2023. "Inference and Reconstruction of the Heimdallarchaeial Ancestry of Eukaryotes." *Nature* 618 (7967): 992–999.

Field, Mark C., and Michael P. Rout. 2019. "Pore Timing: The Evolutionary Origins of the Nucleus and Nuclear Pore Complex." *F1000Research* 8 (April). https://doi.org/10.12688/f1000research.16402.1.

bioRxiv preprint doi: https://doi.org/10.64898/2026.10.28.762692; this version posted October 29, 2026. The copyright holder for this preprint (which was not certified by peer review) is the author/funder, who has granted bioRxiv a license to display the preprint in perpetuity. It is made available under a CC-BY 4.0 International license.

Hartman, Hyman, and Alexei Fedorov. 2002. “The Origin of the Eukaryotic Cell: A Genomic Investigation.” *Proceedings of the National Academy of Sciences of the United States of America* 99 (3): 1420–1425.

Hatano, Tomoyuki, Saravanan Palani, Dimitra Papatziamou, et al. 2022. “Asgard Archaea Shed Light on the Evolutionary Origins of the Eukaryotic Ubiquitin-ESCRT Machinery.” *Nature Communications* 13 (1): 3398.

Hegde, Ramanujan S. 2022. “The Function, Structure, and Origins of the ER Membrane Protein Complex.” *Annual Review of Biochemistry* 91 (June): 651–678.

Huang, Wen-Cong, Maraike Probst, Zheng-Shuang Hua, et al. 2025. “Phylogenomic Analyses Reveal That Panguiarchaeum Is a Clade of Genome-Reduced Asgard Archaea Within the Njordarchaeia.” *Molecular Biology and Evolution* 42 (9). https://doi.org/10.1093/molbev/msaf201.

Imachi, Hiroyuki, Masaru K. Nobu, Nozomi Nakahara, et al. 2020. “Isolation of an Archaeon at the Prokaryote-Eukaryote Interface.” *Nature* 577 (7791): 519–525.

Jumper, John, Richard Evans, Alexander Pritzel, et al. 2021. “Highly Accurate Protein Structure Prediction with AlphaFold.” *Nature* 596 (7873): 583–589.

Kanehisa, Minoru, Yoko Sato, and Masayuki Kawashima. 2022. “KEGG Mapping Tools for Uncovering Hidden Features in Biological Data.” *Protein Science : A Publication of the Protein Society* 31 (1): 47–53.

Kempen, Michel van, Stephanie S. Kim, Charlotte Tumescheit, et al. 2024. “Fast and Accurate Protein Structure Search with Foldseek.” *Nature Biotechnology* 42 (2): 243–246.

Köstlbacher, Stephan, Jolien J. E. van Hooff, Kassiani Panagiotou, et al. 2026. “Prediction of Eukaryotic Cellular Complexity in Asgard Archaea Using Structural Modelling.” *Nature Microbiology* 11 (3): 747–758.

Lewis, Aaron J. O., and Ramanujan S. Hegde. 2021. “A Unified Evolutionary Origin for the Ubiquitous Protein Transporters SecY and YidC.” *BMC Biology* 19 (1): 266.

Lin, Zeming, Halil Akin, Roshan Rao, et al. 2023. “Evolutionary-Scale Prediction of Atomic-Level Protein Structure with a Language Model.” *Science (New York, N.Y.)* 379 (6637): 1123–1130.

Liu, Jiwei, Matteo Tassinari, Diorge P. Souza, et al. 2021. “Bacterial Vipp1 and PspA Are Members of the Ancient ESCRT-III Membrane-Remodeling Superfamily.” *Cell* 184 (14): 3660–3673.e18.

Liu, Yang, Kira S. Makarova, Wen-Cong Huang, et al. 2021. “Expanded Diversity of Asgard Archaea and Their Relationships with Eukaryotes.” *Nature* 593 (7860): 553–557.

López-García, Purificación, and David Moreira. 2020. “The Syntrophy Hypothesis for the Origin of Eukaryotes Revisited.” *Nature Microbiology* 5 (5): 655–667.

Mahendrarajah, Tara A., Edmund R. R. Moody, Dominik Schrempf, et al. 2023. “ATP Synthase Evolution on a Cross-Braced Dated Tree of Life.” *Nature Communications* 14 (1): 7456.

Manni, Mosè, Matthew R. Berkeley, Mathieu Seppey, and Evgeny M. Zdobnov. 2021. “BUSCO: Assessing Genomic Data Quality and Beyond.” *Current Protocols* 1 (12):

bioRxiv preprint doi: https://doi.org/10.64898/2026.10.28.762692; this version posted October 29, 2026. The copyright holder for this preprint (which was not certified by peer review) is the author/funder, who has granted bioRxiv a license to display the preprint in perpetuity. It is made available under a CC-BY 4.0 International license.

e323.

Mantel, N., and W. Haenszel. 1959. “Statistical Aspects of the Analysis of Data from Retrospective Studies of Disease.” *Journal of the National Cancer Institute* 22 (4): 719–748.

Martijn, Joran, Julian Vosseberg, Lionel Guy, Pierre Offre, and Thijs J. G. Ettema. 2018. “Deep Mitochondrial Origin Outside the Sampled Alphaproteobacteria.” *Nature* 557 (7703): 101–105.

Muñoz-Gómez, Sergio A., Edward Susko, Kelsey Williamson, et al. 2022. “Site-and-Branch-Heterogeneous Analyses of an Expanded Dataset Favour Mitochondria as Sister to Known Alphaproteobacteria.” *Nature Ecology & Evolution* 6 (3): 253–262.

Nikov, Georgi I., Daniella Pretorius, and James W. Murray. 2025. “SOLeNNoID: A Deep Learning Pipeline for Solenoid Residue Detection in Protein Structures.” *Bioinformatics (Oxford, England)* 41 (8). https://doi.org/10.1093/bioinformatics/btaf415.

Parks, Donovan H., Maria Chuvochina, Christian Rinke, Aaron J. Mussig, Pierre-Alain Chaumeil, and Philip Hugenholtz. 2022. “GTDB: An Ongoing Census of Bacterial and Archaeal Diversity through a Phylogenetically Consistent, Rank Normalized and Complete Genome-Based Taxonomy.” *Nucleic Acids Research* 50 (D1): D785–D794.

Richardson, Lorna, Ben Allen, Germana Baldi, et al. 2023. “MGnify: The Microbiome Sequence Data Analysis Resource in 2023.” *Nucleic Acids Research* 51 (D1): D753–D759.

Robins, J., N. Breslow, and S. Greenland. 1986. “Estimators of the Mantel-Haenszel Variance Consistent in Both Sparse Data and Large-Strata Limiting Models.” *Biometrics* 42 (2): 311–323.

Rodrigues-Oliveira, Thiago, Florian Wollweber, Rafael I. Ponce-Toledo, et al. 2023. “Actin Cytoskeleton and Complex Cell Architecture in an Asgard Archaeon.” *Nature* 613 (7943): 332–339.

Roger, Andrew J., Sergio A. Muñoz-Gómez, and Ryoma Kamikawa. 2017. “The Origin and Diversification of Mitochondria.” *Current Biology : CB* 27 (21): R1177–R1192.

Schaeffer, R. Dustin, Jimin Pei, Rui Guo, et al. 2026. “Domain Classification of Archaeal Proteomes Reveals Conserved Fold Repertoire.” *PLoS Computational Biology* 22 (9): e1014188.

Spang, Anja, Jimmy H. Saw, Steffen L. Jørgensen, et al. 2015. “Complex Archaea That Bridge the Gap between Prokaryotes and Eukaryotes.” *Nature* 521 (7551): 173–179.

Steinegger, Martin, and Johannes Söding. 2017. “MMseqs2 Enables Sensitive Protein Sequence Searching for the Analysis of Massive Data Sets.” *Nature Biotechnology* 35 (11): 1026–1028.

Steinegger, Martin, and Johannes Söding. 2018. “Clustering Huge Protein Sequence Sets in Linear Time.” *Nature Communications* 9 (1): 2542.

Suzek, Baris E., Yuqi Wang, Hongzhan Huang, Peter B. McGarvey, Cathy H. Wu, and UniProt Consortium. 2015. “UniRef Clusters: A Comprehensive and Scalable Alternative for Improving Sequence Similarity Searches.” *Bioinformatics (Oxford, England)* 31 (6):

bioRxiv preprint doi: https://doi.org/10.64898/2026.10.28.762692; this version posted October 29, 2026. The copyright holder for this preprint (which was not certified by peer review) is the author/funder, who has granted bioRxiv a license to display the preprint in perpetuity. It is made available under a CC-BY 4.0 International license.

926–932.

Tran, Nhan van, Leslie Muller, Robert L. Ross, et al. 2018. “Evolutionary Insights into Trm112-Methyltransferase Holoenzymes Involved in Translation between Archaea and Eukaryotes.” *Nucleic Acids Research* 46 (16): 8483–8499.

Varadi, Mihaly, Damian Bertoni, Paulyna Magana, et al. 2024. “AlphaFold Protein Structure Database in 2024: Providing Structure Coverage for over 214 Million Protein Sequences.” *Nucleic Acids Research* 52 (D1): D368–D375.

Vosseberg, Julian, Jolien J. E. van Hooff, Stephan Köstlbacher, Kassiani Panagiotou, Daniel Tamarit, and Thijs J. G. Ettema. 2024. “The Emerging View on the Origin and Early Evolution of Eukaryotic Cells.” *Nature* 633 (8029): 295–305.

Williamson, Kelsey, Laura Eme, Hector Baños, et al. 2025. “A Robustly Rooted Tree of Eukaryotes Reveals Their Excavate Ancestry.” *Nature* 640 (8060): 974–981.

Williams, Tom A., Cymon J. Cox, Peter G. Foster, Gergely J. Szöllősi, and T. Martin Embley. 2020. “Phylogenomics Provides Robust Support for a Two-Domains Tree of Life.” *Nature Ecology & Evolution* 4 (1): 138–147.

Wright, Erik S. 2025. “Tandem Repeats Provide Evidence for Convergent Evolution to Similar Protein Structures.” *Genome Biology and Evolution* 17 (2). https://doi.org/10.1093/gbe/evaf013.

Yeo, Jingi, Yewon Han, Nicola Bordin, et al. 2025. “Metagenomic-Scale Analysis of the Predicted Protein Structure Universe.” In *bioRxiv*. April 26. https://doi.org/10.1101/2025.04.23.650224.

Yu, Zhaoxi, Lingjie Meng, Canh Hao Nguyen, Hiroshi Mamitsuka, Minoru Kanehisa, and Hiroyuki Ogata. 2026. “DeepKOALA: A Scalable Deep Learning Framework for KEGG Orthology Assignment.” *Briefings in Bioinformatics* 27 (4). https://doi.org/10.1093/bib/bbag445.

Zaremba-Niedzwiedzka, Katarzyna, Eva F. Caceres, Jimmy H. Saw, et al. 2017. “Asgard Archaea Illuminate the Origin of Eukaryotic Cellular Complexity.” *Nature* 541 (7637): 353–358.

Zhang, Jiawei, Xiaoyuan Feng, Meng Li, et al. 2025. “Deep Origin of Eukaryotes Outside Heimdallarchaeia within Asgardarchaeota.” *Nature* 642 (8069): 990–998.

Zhou, Xibin, Chenchen Han, Yingqi Zhang, et al. 2025. “Decoding the Molecular Language of Proteins with Evola.” In *bioRxiv*. BioRxiv. https://doi.org/10.1101/2025.01.05.630192.
